# A prehospital risk assessment tool predicts clinical outcomes in hospitalized patients with heat-related illness: a Japanese nationwide prospective observational study

**DOI:** 10.1038/s41598-023-28498-z

**Published:** 2023-01-21

**Authors:** Ryosuke Takegawa, Jun Kanda, Arino Yaguchi, Shoji Yokobori, Kei Hayashida

**Affiliations:** 1 Department of Traumatology and Acute Critical Medicine, Osaka University Graduate School of Medicine, Osaka, Japan; 2 Department of Emergency Medicine, Teikyo University Hospital, Tokyo, Japan; 3 Department of Critical Care and Emergency Medicine, Tokyo Women’s Medical University, Tokyo, Japan; 4 Department of Emergency and Critical Care Medicine, Nippon Medical School, Tokyo, Japan; 5 Department of Emergency and Critical Care Medicine, School of Medicine, Keio University, Tokyo, Japan; 6 Department of Emergency Medicine, South Shore University Hospital, Northwell Health System, Bay Shore, NY USA; 7 The Feinstein Institutes for Medical Research, Northwell Health System, Manhasset, NY USA

**Keywords:** Diseases, Medical research

## Abstract

We previously developed a risk assessment tool to predict outcomes after heat-related illness (J-ERATO score), which consists of six binary prehospital vital signs. We aimed to evaluate the ability of the score to predict clinical outcomes for hospitalized patients with heat-related illnesses. In a nationwide, prospective, observational study, adult patients hospitalized for heat-related illnesses were registered. A binary logistic regression model and receiver operating characteristic (ROC) curve analysis were used to assess the relationship between the J-ERATO and survival at hospital discharge as a primary outcome. Among eligible patients, 1244 (93.0%) survived to hospital discharge. Multivariable logistic regression analysis revealed that the J-ERATO was an independent predictor for survival to discharge (adjusted odds ratio [OR] 0.47; 95% confidence interval [CI] 0.37–0.59) and occurrence of disseminated intravascular coagulation (DIC) on day 1 (adjusted OR 2.07; 95% CI 1.73–2.49). ROC analyses revealed an optimal J-ERATO cut-off of 5 for prediction of mortality at discharge (area under the curve [AUC] 0.742; 95% CI 0.691–0.787) and DIC development on day 1 (AUC 0.723; 95% CI 0.684–0.758). The J-ERATO obtained before transportation could be helpful in predicting the severity and mortality of hospitalized patients with heat-related illnesses.

## Introduction

Rising global temperatures lead to heat waves, directly causing heat-related illnesses^1,2^. Severe heat waves, such as those that occurred in Europe in 2003, India in 2010, and Japan in 2018, lead to large numbers of patients with heat-related illnesses and high mortality^3–5^. Moreover, an increase in the prevalence of heat-related illness is associated with the increase in the age of the population, as older individuals are more susceptible to a hot environment^6^. During the past 20 years, there has been a 53.7% increase in heat-related mortality in people older than 65 years, reaching a total of 296,000 deaths in 2018 worldwide^7^. Thus, heat-related illnesses are becoming a more common emergency condition with life-threatening consequences. Among heat-related illnesses, heatstroke is the most hazardous condition, in which a person experiences an extremely high core temperature (usually > 40.5 °C), resulting in central nervous system (CNS) dysfunction, multiorgan failure, and disseminated intravascular coagulation (DIC)^8–10^. Previously, we developed a tool, derived using a Japanese nationwide database (in 2010 and 2012) of heat-related illnesses, to predict the disposition and mortality of patients with heat-related illnesses^11^. The early risk assessment tool for detecting clinical outcomes in patients with heat-related illness, called the J-ERATO score, was defined as the sum of six binary components in the prehospital setting (respiratory rate ≥ 22 /min, Glasgow Coma Scale score < 15, systolic blood pressure ≤ 100 mmHg, heart rate ≥ 100 bpm, body temperature ≥ 38 °C, and age ≥ 65 years), with a total score ranging from 0 to 6 (Supplemental Table 1). We demonstrated that the J-ERATO score is accurate in predicting ICU admission and in-hospital mortality^11,12^. Furthermore, the usefulness of the J-ERATO score has been successfully validated in South India, which has a population with a different age distribution to Japan^13^.

Early recognition of and treatment for heatstroke can reduce morbidity and mortality irrespective of its etiology^8,14,15^. Rapid whole-body cooling followed by advanced care in the ICU is essential to improve survival after exertional heatstroke^16–18^. As most cases of heat-related illness are encountered in the prehospital phase in emergency care settings, rapid recognition, and assessment of patients with clinical manifestations of heatstroke in the prehospital setting play pivotal roles in the timely initiation of interventions. The best practices require rapid cooling of the patient to a temperature less than the threshold for critical cell damage (~104.5°F) in less than 30 min from the time of collapse. Cold water immersion is considered the gold standard treatment, but unfortunately, there are many situations in which this method is unavailable^17^. Clinical prediction scores and laboratory measures related to outcomes could be useful to prepare for possible clinical deterioration. However, no precise clinical score or biomarker has been adopted for the prediction of clinical deterioration and prognosis in patients hospitalized for heat-related illnesses. To address this clinical gap, we evaluated the relationship between the J-ERATO score and clinical deterioration after hospital admission in a cohort of patients who required hospitalization for heat-related illnesses.

The purpose of this study was to evaluate the ability of the J-ERATO score, which was obtained by emergency medical service (EMS) personnel at the scene, to predict mortality and the development of DIC and multiorgan dysfunction among patients hospitalized for heat-related illnesses.

## Methods

### Study design and settings

This analysis was conducted using a registered database of a prospective, multicenter, observational study (the Heatstroke Study) in Japan from July 1st to September 30th in 2019, 2020, and 2021. Briefly, the Japanese Association for Acute Medicine (JAAM; Heatstroke and Hypothermia Surveillance Committee) established the Heatstroke Study in 2006. It involved a survey of patients with presumed heat-related illnesses who were transferred to emergency hospitals by EMS personnel^11,19–23^. Since 2017, this registry has been used to gather information regarding only patients admitted to the hospital owing to a diagnosis of heat-related illness. Approximately 142 emergency hospitals from all over Japan took part in the registry during the study period^12^. Data were manually recorded by the staff at each participating hospital by using standardized record sheets. The study protocol was approved by the Teikyo University Ethical Review Board for Medical and Health Research (approval number; 17-021-5, board name; “Heatstroke STUDY”, approval date; May 21st, 2020), which waived the requirement for informed patient consent owing to participant anonymity. The procedures followed were in accordance with the ethical standards of the institution’s responsible committee on human experimentation and with the Helsinki Declaration of 1975, as most recently amended.

### Definition of heat-related illnesses

Heat-related illnesses were defined as conditions in the spectrum of illnesses progressing from heat exhaustion to heatstroke during exercise or exposure to environmental heat stress. Heat-related illness was diagnosed by the attending physician in the emergency department where the patient was admitted.

### Selection of participants

This study included adult patients (aged ≥ 18 years) with a diagnosis of heat-related illness who were admitted to the hospital. The heat-related illness was diagnosed by the modified definition of heatstroke (mJAAM) criteria^19^. Briefly, the mJAAM criteria consist of factors such as CNS manifestations, hepatic/renal dysfunction, and DIC, but not body temperature. Patients who were not directly transferred from the occurrent site to the participant’s hospital, for whom transportation data were missing, or who suffered cardiopulmonary arrest on arrival were excluded from this study. In addition, patients for whom data on the primary outcome, J-ERATO score, event location, and/or the circumstances of the occurrence were missing were excluded from the analyses.

### Data collection

Patient demographics, prehospital information collected by the EMS personnel, and in-hospital information were prospectively recorded (i.e., event location, circumstances of the occurrence, transportation, pre-existing functional dependency, age, sex, prehospital vital signs [Glasgow Coma Scale score, pulse rate, non-invasive blood pressure, respiratory rate, body temperature, and peripheral pulse oximetry], medical history [liver disease, cerebrovascular disease, respiratory disease, chronic kidney disease, immunocompromised disease, psychological disorder, diabetes mellitus with organ dysfunction, and previous heat-related illness], physical findings assessed by the EMS at the scene (seizure, dry skin, skin redness, and skin hotness to touch), in-hospital information [the location of admission, i.e., the ICU or a non-ICU general ward), and survival to hospital discharge. In addition, we gathered information about the patients’ Sequential Organ Failure Assessment (SOFA) score^24^ on the first day after admission (day 1) and the presence of DIC on day 1. Owing to the lack of a specific mortality prediction tool for heat-related illnesses, the SOFA score, which is a general scoring system for critically ill patients, is commonly used to estimate the severity of heat-related illnesses^25^. DIC was diagnosed according to the JAAM DIC diagnostic criteria^26,27^, with a total score ≥ 4 establishing a diagnosis of DIC. Scoring is based on peripheral blood platelet counts, prothrombin time, fibrinogen/fibrin degradation products or D-dimer levels, and the presence of systemic inflammatory response syndrome.

### Outcomes

The primary outcome was survival to hospital discharge. The secondary outcomes were occurrences of DIC and SOFA score on day 1.

### Statistical analysis

Baseline characteristics were summarized using medians and interquartile ranges for continuous variables and frequencies (%) for categorical variables. The differences between groups were tested using the Mann–Whitney U test. The chi-square or Fisher’s exact test was used to compare binary variables. We evaluated the relationship between the J-ERATO and SOFA scores using Spearman's rank correlation test.

Multivariable logistic regression was used to adjust for the selected covariates to determine whether the J-ERATO is related to the prognosis of patients hospitalized for heat-related illnesses. The following independent variables were selected for the model: the J-ERATO score, event location, circumstances of the occurrence, medical history of psychiatric disorder, and age. A set of selected covariates was chosen a priori based on biological plausibility and a priori knowledge^28–32^.

We calculated the areas under the receiver operating characteristic curve (AUCs) and their 95% confidence intervals (CIs) to evaluate the predictive ability of J-ERATO score to differentiate between survival and non-survival at hospital discharge, and between patients with and those without DIC on day 1. The best cut-off point for the J-ERATO score to determine the highest mortality risk was based on Youden’s index. We also performed multivariable analyses in the subgroup of ICU-admitted patients, adjusting for the same variables as in the overall analyses.

All statistical analyses were carried out with a two-sided significance level of 5% via JMP PRO 16.0.0 (http://www.jmp.com).

## Results

### Characteristics of study subjects

**Figure 1:**
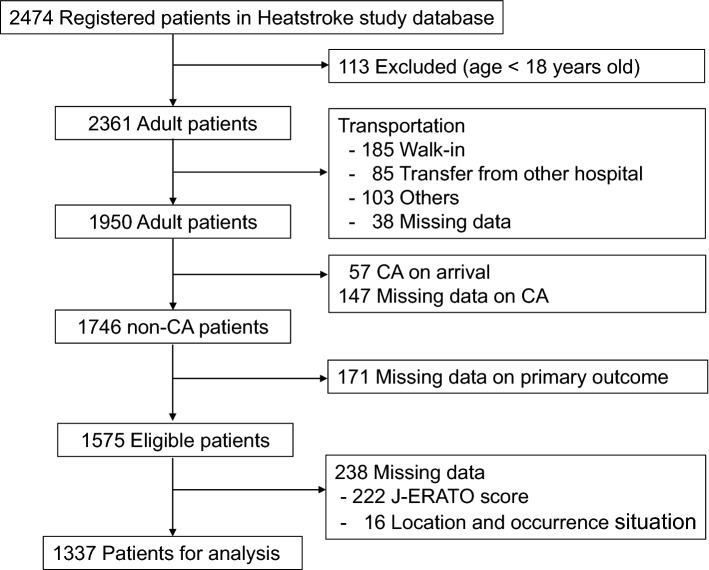
Patient flowchart. CA, cardiac arrest.

A total of 2474 hospitalized patients diagnosed with heat-related illnesses were registered during the study period. After excluding 1137 patients, 1337 patients were analyzed in this study (Fig. 1).

Of the 1337 eligible patients, 1244 (93.0%) survived to hospital discharge. Table 1 summarizes the patient characteristics of the survivor and non-survivor cohorts. Survivors were younger (*P* = 0.03) and more commonly experienced events outdoors (*P* < 0.0001) than non-survivors. Compared with survivors, non-survivors exhibited a significant deterioration of prehospital vital signs, with lower systolic and diastolic blood pressures and SpO_2_, and a higher pulse rate, respiratory rate, body temperature, and disorientation rate (all *P* < 0.0001). Non-survivors had a higher proportion of seizures at the scene than did survivors.

**Table 1:** Patients’ characteristics.

Variable	Overall (n = 1337)	Survivors at discharge (n = 1244)	Non-survivors (n = 93)	*P* value
Male sex, n (%)	906 (68.1)	848 (68.4)	58 (63.0)	0.30
Missing	6 (0.4)	5 (0.4)	1 (1.1)	
Age, years old, median [IQR]	76 [63–85]	75.5 [62–84]	79 [67–86]	0.03
Missing	0 (0)	0 (0)	0 (0)	
Body mass index, kg/m^2^, median [IQR]	22 [19.1–24.9]	22.0 [19.0–24.8]	22.5 [19.5–25.8]	0.28
Missing	240 (18.0)	221 (17.8)	19 (20.4)	
Event location, n (%)				< 0.0001
Outside	649 (48.5)	623 (50.1)	26 (28.0)	
Indoor	688 (51.5)	621 (49.9)	67 (72.0)	
Missing	0 (0)	0 (0)	0 (0)	
Occurrence situation, n (%)				0.0004
Physical labour	330 (24.7)	322 (25.9)	8 (8.6)	
Office work	9 (0.7)	8 (0.6)	1 (1.1)	
Sports	38 (2.8)	38 (3.1)	0 (0)	
Daily life	960 (71.8)	876 (70.4)	84 (90.3)	
Missing	0 (0)	0 (0)	0 (0)	
Past medical history, n (%)				
Liver disease	33 (2.5)	31 (2.6)	2 (2.2)	1
Missing	42 (3.1)	39 (3.1)	3 (3.2)	
Cerebrovascular disease	89 (6.8)	81 (6.7)	8 (9.0)	0.38
Missing	36 (2.7)	32 (2.6)	4 (4.3)	
Respiratory disease	44 (3.4)	41 (3.4)	3 (3.4)	1
Missing	43 (3.2)	39 (3.1)	4 (4.3)	
Kidney disease	24 (1.9)	23 (1.9)	1 (1.1)	1
Missing	45 (3.4)	41 (3.3)	4 (4.3)	
Immunocompromised disease	36 (2.8)	33 (2.7)	3 (3.4)	0.73
Missing	44 (3.4)	39 (3.1)	5 (5.4)	
Psychiatric disorder	165 (12.7)	153 (12.6)	12 (13.3)	0.87
Missing	34 (2.5)	31 (2.5)	3 (3.2)	
Diabetes with organ dysfunction	44 (3.4)	40 (3.3)	4 (4.5)	0.54
Missing	47 (3.5)	43 (3.5)	4 (4.3)	
Previous heat illness	76 (5.9)	72 (6.0)	4 (4.6)	0.81
Missing	53 (4.0)	47 (3.8)	6 (6.5)	
Vital signs at the scene				
Prehospital SBP, mmHg, median [IQR]	127 [101–150]	129 [104–151]	94 [72–125]	< 0.0001
Unmeasurable, n (%)	0 (0)	0 (0)	0 (0)	
Missing, n (%)	0 (0)	0 (0)	0 (0)	
Prehospital DBP, mmHg, median [IQR]	74 [60–88]	75 [60–89]	54 [43–81]	< 0.0001
Unmeasurable, n (%)	8 (0.6)	7 (0.6)	1 (1.1)	
Missing, n (%)	49 (3.7)	41 (3.3)	8 (8.6)	
Prehospital PR, bpm, median [IQR]	113 [93–130]	112 [91–129]	121[110–141]	< 0.0001
Unmeasurable, n (%)	0 (0)	0 (0)	0 (0)	
Missing, n (%)	0 (0)	0 (0)	0 (0)	
Prehospital RR/ min, median [IQR]	24 [20–30]	24 [20–30]	30 [24–36]	< 0.0001
Unmeasurable, n (%)	0 (0)	0 (0)	0 (0)	
Missing, n (%)	0 (0)	0 (0)	0 (0)	
Prehospital SpO_2_, %, median [IQR]	96 [93–98]	96 [93–98]	92 [88–96]	< 0.0001
Unmeasurable, n (%)	16 (1.2)	9 (0.7)	7 (7.5)	
Missing, n (%)	18 (1.3)	18 (1.4)	0 (0)	
Prehospital BT, °C, median [IQR]	38.9 [37.2–40.2]	38.8 [37.1–40.1]	39.8 [38.6–40.8]	< 0.0001
Unmeasurable, n (%)	0 (0)	0 (0)	0 (0)	
Missing, n (%)	0 (0)	0 (0)	0	
Prehospital GCS category, n (%)				< 0.0001
GCS = 15	253 (18.9)	253 (20.3)	0 (0)	
GCS < 15	1084 (81.1)	991 (79.7)	93 (100)	
Missing	0 (0)	0 (0)	0 (0)	
Physical findings at the scene				
Seizure, n (%)	120 (9.2)	109 (9.0)	11 (12.0)	0.016
Unknown	48 (3.7)	40 (3.3)	8 (8.7)	
Missing	34 (2.5)	33 (2.7)	1 (1.1)	
Dry skin, n (%)	402 (33.3)	376 (33.4)	26 (32.1)	0.55
Unknown	471 (39.0)	438 (38.9)	33 (40.7)	
Missing	129 (9.6)	117 (9.4)	12 (12.9)	
Skin redness, n (%)	216 (17.7)	197 (17.4)	19 (22.1)	0.55
Unknown	419 (34.4)	391 (34.6)	28 (32.6)	
Missing	120 (9.0)	113 (9.1)	7 (7.5)	
Skin hotness to touch, n (%)	594 (47.9)	546 (47.4)	48 (55.2)	0.32
Unknown	336 (27.1)	314 (27.2)	22 (25.3)	
Missing	97 (7.3)	91 (7.3)	6 (6.5)	

IQR, Interquartile range; SBP, systolic blood pressure; DBP, diastolic blood pressure; PR, pulse rate; RR, respiratory rate; BT, body temperature; GCS, Glasgow coma scale.

Table 2 summarizes the duration of hospitalization, outcomes, and severity scores among the survivors and non-survivors. Survivors had a lower proportion of ICU admission (*P* < 0.0001) than non-survivors. Among the patients admitted to ICU (560 of 1337 eligible patients [41.9%]), survivors had significantly longer ventilator-free (*P* < 0.0001) and ICU-free (*P* < 0.0001) days than non-survivors. On day 1, fewer survivors had DIC (*P* < 0.0001), and survivors had lower SOFA scores (*P* < 0.0001) than non-survivors.

**Table 2:** Duration of hospitalization, outcomes, and severity scores between survivors and non-survivors.

Variable	Overall (n = 1337)	Survivors (n = 1244)	Non-survivors (n = 93)	*P* value
J-ERATO score, median [IQR]	4 [3–5]	4 [2–5]	5 [4–6]	< 0.0001
Missing, n (%)	0 (0)	0 (0)	0 (0)	
Duration of hospital days, median [IQR]	6 [3–15]	6 [3–15]	5 [2–14.75]	0.28
Missing, n (%)	6 (0.5)	5 (0.4)	1 (1.1)	
ICU admission, n (%)	560 (42.5)	499 (40.7)	61 (70.9)	< 0.0001
Missing, n (%)	20 (1.5)	18 (1.4)	2 (2.3)	
Ventilator free days, median [IQR]	5 [2–15]	6 [3–16]	1 [0–6.5]	< 0.0001
Missing, n (%)	121 (9.1)	113 (9.1)	8 (8.6)	
ICU free days, median [IQR]	5 [2–14]	5 [2–15]	0 [0–5]	< 0.0001
Missing, n (%)	93 (7.0)	84 (6.8)	9 (9.7)	
Presence of DIC on day 1, n (%)	184 (22.3)	148 (19.5)	36 (56.3)	< 0.0001
Missing, n (%)	512 (38.3)	483 (38.8)	29 (31.2)	
DIC score on day1, median [IQR]	1 [1–3]	1 [1–3]	4 [2–5]	< 0.0001
Missing, n (%)	552 (41.3)	520 (41.8)	32 (34.4)	
SOFA score on day1, median [IQR]	5 [3–7]	4 [3–6]	10 [7–12]	< 0.0001
Missing, n (%)	400 (29.9)	383 (30.8)	17 (18.3)	

IQR, interquartile range; ICU, intensive care unit; DIC, disseminated intravascular coagulation; SOFA, sequential organ failure assessment.

### Main results

Table 3 demonstrates the association between the J-ERATO score and primary outcome among all patients. The J-ERATO score was an independent negative predictor of survival at hospital discharge. Table 4 demonstrates the association between the J-ERATO score and the occurrence of DIC among all patients. The J-ERATO score was an independent positive predictor of DIC on day 1.

**Table 3:** Association of survival at hospital discharge with the J-ERATO score among all study subjects.

	Crude OR (95% CI)	Adjusted OR (95% CI)	*P* value
J-ERATO score	0.46 (0.37–0.57)	0.47 (0.37–0.59)	< 0.0001
Location (outside)	2.59 (1.62–4.12)	2.02 (1.19–3.43)	0.0009
Occurrence status (exertion)	4.33 (2.07–9.02)	2.16 (0.94–4.96)	0.069
Psychiatric disorder	0.94 (0.50–1.76)	1.18 (0.60–2.32)	0.63
Age	0.98 (0.97–1.00)	1.01 (1.00–1.03)	0.11

Logistic regression models were used with adjustment for J-ERATO score, event location, circumstances of the occurrence, medical history of psychiatric disorder, and age.

OR, odds ratio; CI, confidence interval.

**Table 4:** Association of presence of DIC on day 1 with the J-ERATO score among all study subjects.

	Crude OR (95% CI)	Adjusted OR (95% CI)	*P* value
J-ERATO score	2.15 (1.80–2.56)	2.07 (1.73–2.49)	< 0.0001
Location (outside)	0.32 (0.23–0.46)	0.42 (0.27–0.62)	< 0.0001
Occurrence status (exertion)	0.23 (0.14–0.38)	0.55 (0.30–0.97)	0.044
Psychiatric disorder	0.63 (0.36–1.05)	0.57 (0.31–1.00)	0.058
Age	1.03 (1.02–1.05)	1.01 (0.99–1.02)	0.46

Logistic regression models were used with adjustment for J-ERATO score, event location, circumstances of the occurrence, medical history of psychiatric disorder, and age.DIC, disseminated intravascular coagulation; OR, odds ratio; CI, confidence interval.

We assessed the heterogeneity of our results by conducting subgroup analyses of ICU-admitted patients. The subgroup results were similar to the main results, emphasizing the role of the J-ERATO score on both survival outcome at discharge and development of DIC on day 1 (Supplemental Table 2).

**Figure 2:**
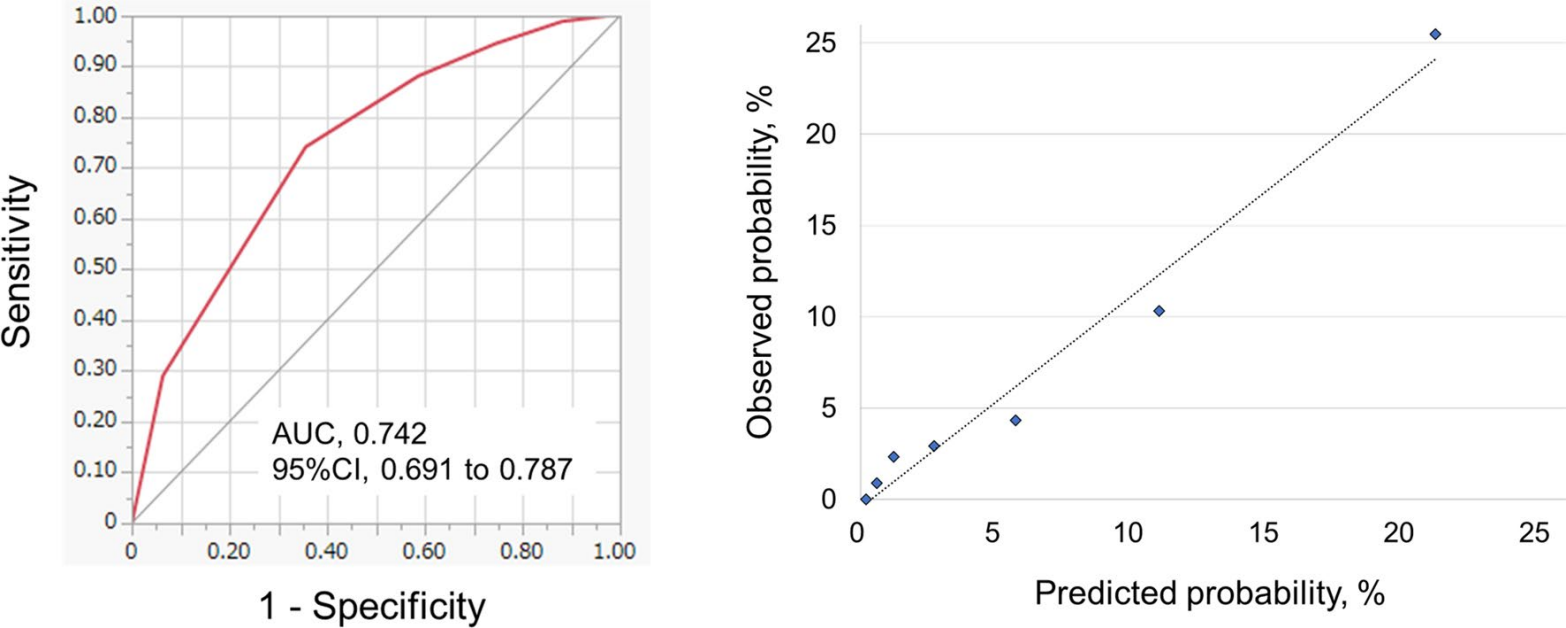
The area under the receiver operating characteristic curve of the J-ERATO score to predict mortality after heat-related illness. (**A**, left) The J-ERATO score cut-off of 5 points provided optimal sensitivity and specificity to predict mortality at discharge (AUC [95%CI], 0.742 [0.691–0.787]; sensitivity, 74.2% [64.5–82.0]; specificity, 64.4% [61.7–67.0]; PPV, 13.5% [10.8–16.7]; NPV, 97.1% [95.7–98.0]; positive likelihood ratio, 2.1 [1.8–2.4]; negative likelihood ratio, 0.4 [0.3–0.6]). (**A**, right) Calibration plot. Left: X-axis: predicted probability, Y-axis: observed probability. AUC, the area under the receiver operating characteristic curve; CI, confidence interval; DIC, disseminated intravascular coagulation; PPV, positive predictive values; NPV, negative predictive values.

We further evaluated the sensitivity and specificity of J-ERATO score cut-off values for the entire cohort. The receiver operating characteristic analyses revealed a J-ERATO score cut-off of 5 points, providing optimal sensitivity and specificity to predict mortality at discharge (AUC, 0.742; 95% CI 0.691–0.787; sensitivity, 74.2%; specificity, 64.4%) (Fig. 2, left). For calibration, the predicted probability and actual observation by score are illustrated (Fig. 2, right).

**Figure 3:**
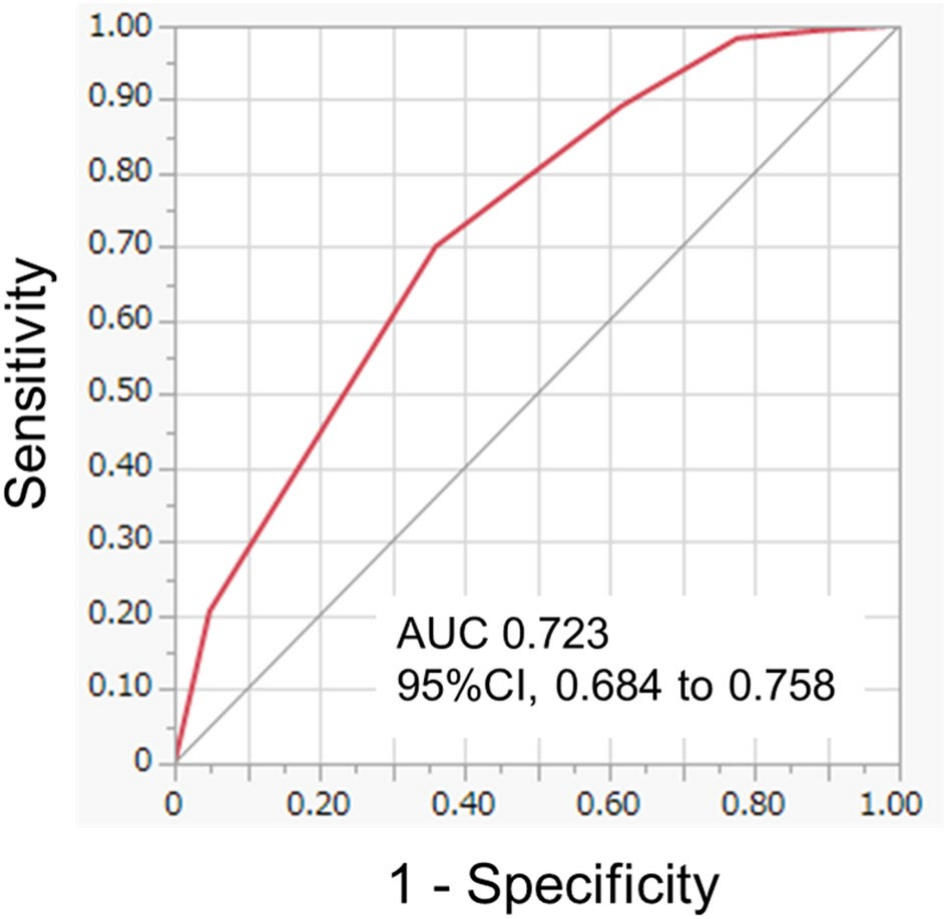
The area under the receiver operating characteristic curve of the J-ERATO score to predict the presence of DIC on day 1. The J-ERATO score cut-off of 5 points provided optimal sensitivity and specificity to predict the presence of DIC on day 1 (AUC [95%CI], 0.723 [0.684–0.758]; sensitivity, 70.1% [63.1–76.3]; specificity, 64.0% [60.2–67.6]; PPV, 35.8% [31.1–40.9]; NPV, 88.2% [84.9–90.8]; positive likelihood ratio, 1.9 [1.7–2.2]; negative likelihood ratio,0.5 [0.4–0.6]). AUC, the area under the receiver operating characteristic curve; CI, confidence interval; DIC, disseminated intravascular coagulation; PPV, positive predictive values; NPV, negative predictive values.

The J-ERATO score cut-off of 5 points also provided optimal sensitivity and specificity to predict DIC development on day 1 (AUC 0.723; 95% CI 0.684–0.758; sensitivity, 70.1%; specificity, 64.0%) (Fig. 3).

**Figure 4:**
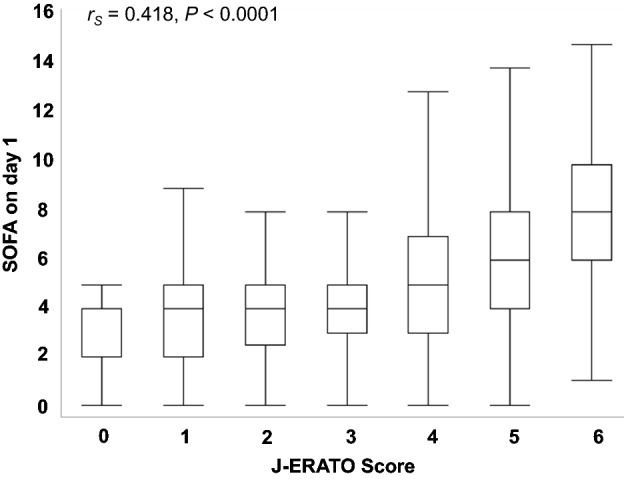
Boxplots displaying the association between the J-ERATO and SOFA scores on the first day after hospital admission. In total, 937 patients were included in the analysis. The numbers of patients in the J-ERATO score groups of 0, 1, 2, 3, 4, 5, and 6 were 19, 75, 97, 140, 224, 302, and 80, respectively. A significant linear trend was observed between the J-ERATO and SOFA scores on day 1 (Spearman’s rank correlation coefficient [*r*_*S*_] = 0.418, *P* < 0.0001). J-ERATO, early risk assessment tool for detecting clinical outcomes in patients with heat-related illness; SOFA: Sequential Organ Failure Assessment.

In this study, a large amount SOFA score data was missing, both in the entire cohort (29.9% [400 of 1337]) and the ICU-admitted cohort (missing, 25.0% [140 of 560]). Figure 4 shows the association between the J-ERATO and SOFA scores on day 1 in the entire cohort. An increased J-ERATO score was significantly associated with an increase in the SOFA score in both cohorts (both *P* < 0.0001). There was a positive association between the J-ERATO and SOFA scores on day 1 (Spearman’s rank correlation coefficient, 0.418; *P* < 0.0001). Subgroup analysis of ICU-admitted patients revealed a similar association between the J-ERATO and SOFA scores (Supplemental Figure 1).

We addressed the issue of missing SOFA score data by conducting additional analyses. Accordingly, there were no differences in age, event location, occurrence circumstances, prehospital blood pressure, ICU admission rate, or mortality between patients with and those without SOFA data. Patients without SOFA data had a lower body temperature, a lower proportion of prehospital CNS abnormalities, and a lower incidence of ICU admissions (Supplemental Table 3).

## Discussion

This study verified the clinical usefulness of the J-ERATO score^11^ for patients with heat-related illnesses who were transferred to the hospital by EMS personnel. To the best of our knowledge, this is the first study to demonstrate that the J-ERATO score is an independent predictive marker for survival to discharge as well as the occurrence of DIC on day 1, and that there is a significantly positive correlation between the J-ERATO score and the severity of organ dysfunction (the SOFA score) on day 1. Given that the J-ERATO score is calculated with only physical information regarding prehospital vital signs and patient age, our observations are very important to improve outcomes in patients with heat-related illnesses.

While risk factors for mortality have been investigated in patients admitted to the ICU with heatstroke^33–35^, few studies have focused on early screening tools for the prediction of clinical outcomes in patients with heat-related illnesses in the prehospital setting, emphasizing the importance of the J-ERATO score. Furthermore, the J-ERATO score has been validated in South India^13^. Ninan et al. reported that a higher J-ERATO score at hospital presentation was an independent predictor of underlying multiple organ dysfunction syndromes, defined as a SOFA score > 2 points (*P* < 0.029)^13^. On the one hand, we previously reported the potential of machine learning-based mortality prediction models for heat-related illnesses (AUC, 0.92), using 24 variables at hospital arrival^22^. However, at present, such a model is not clinically feasible in most cases where heat-related illnesses are encountered, as machine learning algorithms require certain computer equipment for calculation. In general, a cumbersome tool is unlikely to be used consistently by busy physicians. Thus, a simple bedside tool for use at the time of presentation of heat-related illness is necessary. Furthermore, there is strong evidence that immediate and aggressive cooling after heatstroke ensures survival with limited sequelae^8,15,16,36–38^, highlighting the need for appropriate prehospital care. Aggressive treatment in the prehospital setting according to the severity of heat-related illness, as determined with the J-ERATO score, is a promising strategy.

Although no universally accepted definition of heatstroke exists in clinical settings, Bouchama's definition is most commonly used globally^9^. They defined heatstroke as severe illness characterized by a core temperature of > 40 °C and CNS abnormalities resulting from exposure to environmental heat (classic heatstroke) or strenuous physical exercise (exertional heatstroke). However, mortality in heat-related illness is attributable not only to CNS abnormalities but also to organ dysfunction. Of note, the maximum body temperature recorded upon hospital admission after EMS transferal in several fatal cases was below 40 °C^19^. Mortality after heat-related illness increased significantly to approximately 5% at body temperatures above 38.1 °C^19^. To address this clinical weak point in Bouchama's definition, the JAAM first established criteria for heat-related illnesses in 2014 and modified the definition of heatstroke (mJAAM) in 2016^19^. Apart from body temperature, the mJAAM criteria for heatstroke include all the components of Bouchama’s criteria^21^. Kondo et al. used the previous JAAM heatstroke database in 2014 and evaluated the differences between Bouchama’s criteria and mJAAM criteria in terms of diagnosis and identification of mortality. A total of 317 patients were included and divided into the Bouchama, mJAAM, and non-heatstroke groups, each consisting of 97, 302, and 15 patients, respectively. The sensitivity for death was 1.0 (95% CI 0.87–1.0) with the mJAAM criteria and only 0.29 (95% CI 0.14–0.49) with Bouchama’s criteria. On the other hand, both the Bouchama and mJAAM criteria could not predict in-hospital mortality (AUC: 0.52 for both criteria). The median SOFAs were 5 and 3 in the Bouchama and mJAAM criteria, respectively^21^. Taken together with our current results, the diagnosis of heatstroke with the mJAAM criteria in combination with prognostication with the J-ERATO score, especially more than 5 points of J-ERATO score, could enable physicians to optimize treatment and allocate proper medical resources to patients with heat-related illnesses.

DIC has a reported incidence of ≥ 48% among patients with heatstroke^10,39,40^. In the current study, DIC was diagnosed based on the JAAM DIC diagnostic criteria, with a total score ≥ 4 indicating a diagnosis of DIC^26,41^. Gando et al. reported that the JAAM DIC scoring system exhibited superior prognostic value over the International Society on Thrombosis and Haemostasis' overt DIC scoring system for the prediction of multiple organ dysfunction syndromes and poor prognoses in patients with severe sepsis^41^. Hifumi et al. reported that mortality worsened significantly as the JAAM DIC score increased and was approximately 10% even at a DIC score of only 2 in patients with heatstroke according to the mJAAM criteria^19^. In the current study, higher J-ERATO scores were associated with a higher prevalence of DIC and mortality. The AUC of the J-ERATO cut-off of 5 was greater than 0.7, which is regarded as an acceptable predictive accuracy^42^. Taken together, early evaluation of the patient’s status should be helpful in evaluating the severity of heatstroke and determining the optimal treatment at the patient’s presentation.

The current study had several limitations. First, 594/2474 (24.0%) patients in the registry were excluded from the analyses because of missing data, which may confer a risk of bias and affect our results. Especially, the high percentage of missing data may have affected the results of the AUC value. Further evaluation will be needed. Further, 512/1337 (38.3%) patients had missing data regarding the occurrence of DIC and 400/1337 (29.9%) regarding the SOFA score on day 1, for unknown reasons. We addressed the latter issue by comparing parameters between patients with and those without SOFA score data. Our results indicate that the usefulness of the J-ERATO score may be limited to patients with more severe heat-related illness. Second, we did not impute missing values because of the large amount of missing data that would need to be imputed, which might have generated bias. Thus, our findings should be interpreted with caution. Third, although neurological sequelae after heatstroke are clinically important complications^43^, we were not able to analyze the association between the J-ERATO score and neurological dysfunction because of the small number of events. Thus, the role of the J-ERATO score on neurocognitive function after heat-related illness remains undetermined. Fourth, although data were collected prospectively in this study, the J-ERATO score was analyzed in a retrospective manner. Therefore, we could not evaluate the usefulness of the treatment strategy according to the J-ERATO score. In particular, patients were treated according to the discretion of the physician in charge, which may have been based on clinical information, including the J-ERATO score, resulting in bias.

We found that the J-ERATO score has the potential to predict patients' severity expressing after transportation to the hospitals. Therefore, it is expected that EMS personnel calculate the J-ERATO score and start first aid, including aggressive body temperature control. On the other hand, the reconstitution of the database system to decrease the missing data may be needed in the future. Based on the new database, re-evaluating the accuracy of J-ERATO score for the outcomes despite patients’ severity would be desirable. Further, the construction of new transportation systems or prehospital treatment strategies according to the J-ERATO score needs to be considered.

## Conclusion

The J-ERATO score which can be determined by EMS personnel at the scene was an independent predictor of survival at hospital discharge and DIC incidence on day 1 and was positively associated with more severe organ dysfunction on day 1. The J-ERATO score may be helpful in predicting the severity and mortality of patients with heat-related illnesses.

## Supplementary Information

Supplementary Information.

## Data Availability

The datasets used and/or analyzed during the current study available from the corresponding author on reasonable request.
