# Supplementary material for: A prehospital risk assessment tool predicts clinical outcomes in hospitalized patients with heat-related illness: a Japanese nationwide prospective observational study
Source: Sci Rep. 2023 Jan 21;13:1189. doi: 10.1038/s41598-023-28498-z (PMC9867691; doi:10.1038/s41598-023-28498-z)
Supplement: Supplementary file 1 — Supplementary Information. [file 41598_2023_28498_MOESM1_ESM.docx]

**SUPPLEMENTAL MATERIALS**

**A prehospital risk assessment tool predicts clinical outcomes in hospitalized patients with heat-related illness: a Japanese nationwide prospective observational study**

Ryosuke Takegawa, Jun Kanda, Arino Yaguchi, Shoji Yokobori, and Kei Hayashida.

| J-ERATO score |  | Point | |
| --- | --- | --- | --- |
| Variables at prehospital setting |  | 0 | 1 |
| Respiratory Rate (beat/min) |  | < 22 | ≥ 22 |
| Glasgow Coma Scale |  | 15 | ≤ 14 |
| Systolic Blood Pressure (mmHg) |  | > 100 | ≤ 100 |
| Heart Rate (bpm) |  | < 100 | ≥ 100 |
| Body Temperature (ºC) |  | < 38 | ≥ 38 |
| Age (years old) |  | < 65 | ≥ 65 |

**Supplemental Table 1. J-ERATO score**

**Supplemental Table 2. Association of outcomes with the J-ERATO score in a subgroup of ICU admitted patients.**

|  | **Crude OR (95 % CI)** | **Adjusted OR (95 % CI)** | **P value** |
| --- | --- | --- | --- |
| *Survival at hospital discharge* |  |  |  |
| J-ERATO score | 0.57 [0.42-0.76] | 0.53 [0.37-0.73] | 0.0003 |
| Location (outside) | 2.16 [1.25-3.86] | 1.95 [1.05-3.76] | 0.04 |
| Occurrence status (exertion) | 2.66 [1.26-6.55] | 1.82 [0.75-4.93] | 0.21 |
| Psychiatric disorder | 0.98 [0.49-2.11] | 1.18 [0.56-2.67] | 0.68 |
| Age | 1.00 [0.98-1.01] | 1.02 [1.00-1.04] | 0.03 |
|  |  |  |  |
| *Presence of DIC on day 1* |  |  |  |
| J-ERATO score | 2.15 [1.80-2.56] | 1.60 [1.21-2.10] | 0.0009 |
| Location (outside) | 0.32 [0.23-0.46] | 0.36 [0.21-0.61] | 0.0001 |
| Occurrence status (exertion) | 0.23 [0.14-0.38] | 0.53 [0.25-1.14] | 0.10 |
| Psychiatric disorder | 0.63 [0.36-1.05] | 0.49 [0.24-1.02] | 0.06 |
| Age | 1.03 [1.02-1.05] | 1.01 [0.99-1.03] | 0.20 |

Logistic regression models were used with adjustment for J-ERATO score, event location, circumstances of the occurrence, medical history of psychiatric disorder, and age.

ICU, intensive care unit; DIC, disseminated intravascular coagulation; OR, odds ratio; CI, confidence interval.

**
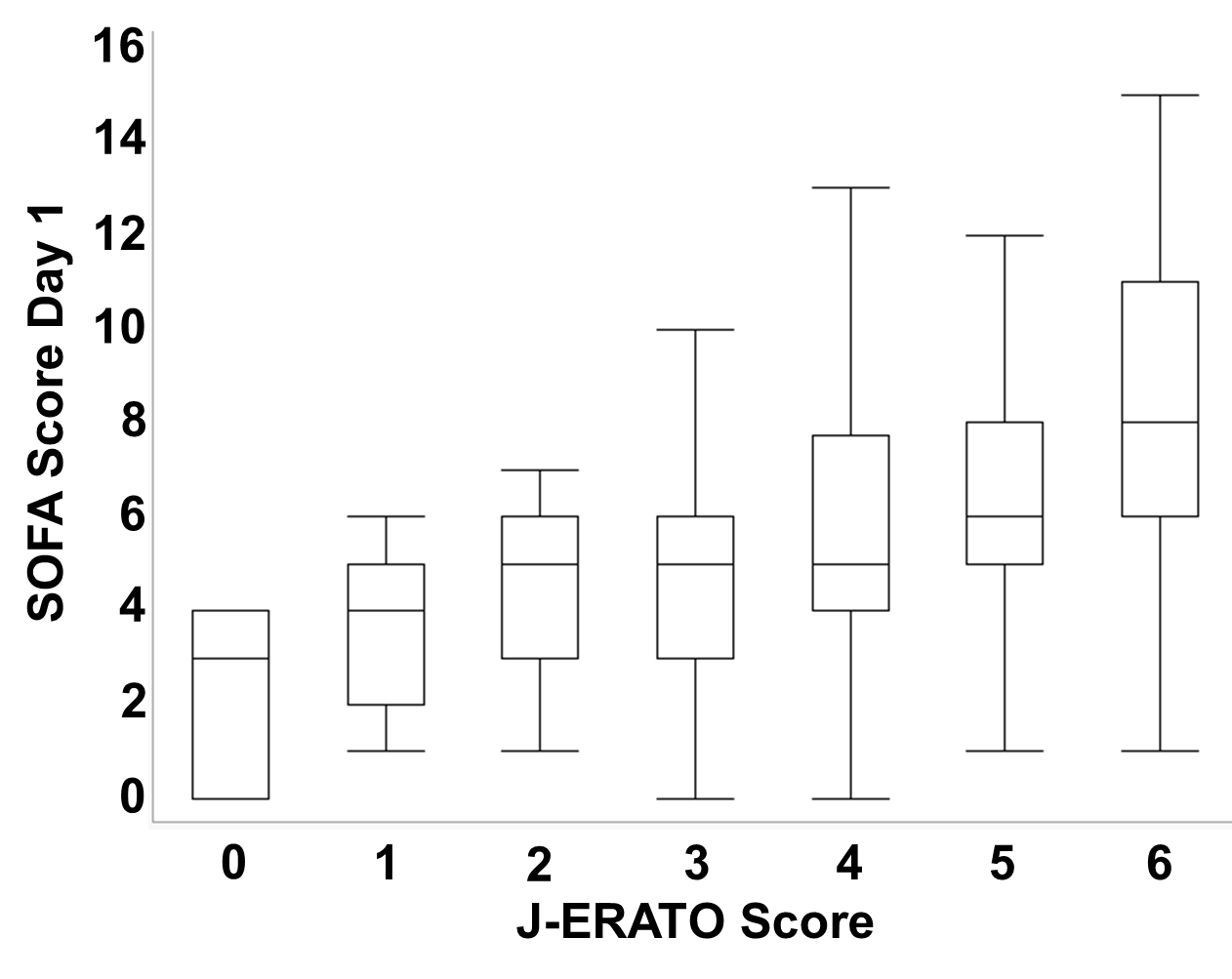
**

**Supplemental Figure 1. Boxplots with dot plots displaying the association between the J-ERATO and SOFA scores on the first day after hospital admission in patients admitted to the ICU.**

In total, 420 patients admitted to the ICU were included in the analysis. The numbers of patients in the J-ERATO score groups of 0, 1, 2, 3, 4, 5, and 6 were 3, 12, 17, 43, 112, 178, and 55, respectively. J-ERATO, early risk assessment tool for detecting clinical outcomes in patients with heat-related illness; SOFA: Sequential Organ Failure Assessment.

**Supplemental Table 3. Patients’ characteristics,** **duration of hospitalization, outcomes, and severity scores between patients with and without data for SOFA score.**

| Variable | Patients with data for SOFA score  (n = 937) | Patients missing data for SOFA score (n = 400) | *P* value |
| --- | --- | --- | --- |
| Male sex, n (%) | 653 (70.1) | 253 (63.4) | 0.018 |
| Missing | 5 (0.5) | 1 (0.3) |  |
| Age, years old, median [IQR] | 76 [63-85] | 74.5 [63-84] | 0.42 |
| Missing | 0 | 0 |  |
| Body mass index, kg/m^2^, median [IQR] | 22.0 [19.0-25.0] | 22.0 [19.5-24.8] | 0.73 |
| Missing | 158 (16.9) | 83 (20.8) |  |
| Event location, n (%) |  |  | 0.68 |
| Outside | 451 (48.1) | 198 (49.5) |  |
| Indoor | 486 (51.9) | 202 (50.5) |  |
| Missing | 0 | 0 |  |
| Occurrence situation, n (%) |  |  | 0.60 |
| Physical labour | 222 (23.7) | 108 (27.0) |  |
| Office work | 6 (0.6) | 3 (0.8) |  |
| Sports | 26 (2.8) | 12 (3.0) |  |
| Daily life | 683 (72.9) | 277 (69.3) |  |
| Missing | 0 | 0 |  |
| Preexisting functional dependency, n (%) |  |  |  |
| Not disabled | 26 (2.8) | 7 (1.9) | 0.34 |
| Disabled | 21 (2.2) | 21 (5.3) |  |
| Missing | 55 (6.0) | 34 (8.9) | 0.07 |
| Past medical history, n (%) | 20 (2.1) | 16 (4.0) |  |
| Liver disease | 29 (3.2) | 15 (3.9) | 0.50 |
| Missing | 26 (2.8) | 17 (4.3) |  |
| Cerebrovascular disease | 14 (1.5) | 10 (2.6) | 0.18 |
| Missing | 25 (2.7) | 20 (5.0) |  |
| Respiratory disease | 19 (2.1) | 17 (4.5) | 0.02 |
| Missing | 24 (2.6) | 20 (5.0) |  |
| Kidney disease | 113 (12.3) | 52 (13.5) | 0.52 |
| Missing | 18 (1.9) | 16 (4.0) |  |
| Immunocompromised disease | 28 (3.1) | 16 (4.2) | 0.32 |
| Missing | 28 (3.0) | 19 (4.8) |  |
| Psychiatric disorder | 49 (5.4) | 27 (7.2) | 0.24 |
| Missing | 30 (3.2) | 23 (5.8) |  |
| Diabetes with organ dysfunction |  |  | 0.60 |
| Missing | 222 (23.7) | 108 (27.0) |  |
| Previous heat illness | 6 (0.6) | 3 (0.8) |  |
| Missing | 26 (2.8) | 12 (3.0) |  |
| Vital signs at the scene |  |  |  |
| Prehospital SBP, mmHg, median [IQR] | 126 [100-150] | 129 [105.3-150] | 0.19 |
| Unmeasurable, n (%) | 0 | 0 |  |
| Missing, n (%) | 0 | 0 |  |
| Prehospital DBP, mmHg, median [IQR] | 73 [60-89] | 74 [60-88] | 0.80 |
| Unmeasurable, n (%) | 8 (0.9) | 0 |  |
| Missing, n (%) | 31 (3.3) | 18 (4.5) |  |
| Prehospital PR, bpm, median [IQR] | 114 [96-132] | 108 [89.3-126] | < 0.0001 |
| Unmeasurable, n (%) | 0 | 0 |  |
| Missing, n (%) | 0 | 0 |  |
| Prehospital RR/ min, median [IQR] | 24 [20-30] | 24 [20-30] | 0.016 |
| Unmeasurable, n (%) | 0 | 0 |  |
| Missing, n (%) | 0 | 0 |  |
| Prehospital SpO_2_, %, median [IQR] | 96 [94-98] | 95 [93-97] | 0.008 |
| Unmeasurable, n (%) | 13 (1.4) | 3 (0.8) |  |
| Missing, n (%) | 3 (0.3) | 10 (2.5) |  |
| Prehospital BT, °C, median [IQR] | 39.0 [37.5-40.3] | 38.5 [36.9-39.8] | < 0.0001 |
| Unmeasurable, n (%) | 0 | 0 |  |
| Missing, n (%) | 0 | 0 |  |
| Prehospital GCS category, n (%) |  |  | < 0.0001 |
| GCS = 15 | 150 (16.0) | 103 (25.8) |  |
| GCS < 15 | 787 (84.0) | 297 (74.3) |  |
| Missing | 0 | 0 |  |
| Physical findings at the scene |  |  |  |
| Seizure, n (%) | 86 (9.3) | 34 (9.0) | 0.08 |
| Unknown | 41 (4.4) | 7 (1.9) |  |
| Missing | 12 (1.3) | 22 (5.5) |  |
| Dry skin, n (%) | 267 (29.9) | 135 (42.7) | < 0.0001 |
| Unknown | 392 (44.0) | 79 (25.0) |  |
| Missing | 45 (4.8) | 84 (21.0) |  |
| Skin redness, n (%) | 163 (18.2) | 53 (16.5) | < 0.0001 |
| Unknown | 345 (38.5) | 74 (23.1) |  |
| Missing | 41 (4.4) | 79 (19.8) |  |
| Skin hotness to touch, n (%) | 453 (50.3) | 141 (41.5) | < 0.0001 |
| Unknown | 272 (30.2) | 64 (18.8) |  |
| Missing | 37 (3.9) | 60 (15.0) |  |
| ICU admission, n (%) | 423 (45.5) | 142 (36.6) | 0.003 |
| Missing, n (%) | 8 (0.9) | 12 (3.0) |  |
| Duration of hospital days, median [IQR] | 6 [3-15] | 5 [2-13] | 0.03 |
| Missing, n (%) | 4 (0.4) | 2 (0.5) |  |
| ICU free days, median [IQR] | 5 [2-13] | 5 [2-19] | 0.06 |
| Missing, n (%) | 53 (5.7) | 40 (10.0) |  |
| Ventilator free days, median [IQR] | 6 [2-15] | 5 [2-20.3] | 0.86 |
| Missing, n (%) | 71 (7.6) | 50 (12.5) |  |
| Presence of DIC on day 1, n (%) | 137 (22.5) | 47 (21.8) | 0.85 |
| Missing, n (%) | 328 (35.0) | 184 (46.0) |  |
| DIC score on day1, median [IQR] | 1 [1-3] | 1 [0-3] | 0.11 |
| Missing, n (%) | 334 (35.6) | 218 (54.5) |  |
| SOFA score day1, median [IQR] | 5 [3-7] | N/A |  |
| Missing, n (%) | 0 | 400 (100) |  |
| 28-day mortality, n (%) | 15 (13.9) | 9 (6.6) | 0.082 |
| Missing, n (%) | 829 (88.5) | 264 (66.0) |  |
| Survival Discharge, n (%) | 861 (91.9) | 383 (95,8) | 0.010 |
| Missing, n (%) | 0 | 0 |  |

IQR, Interquartile range; SBP, systolic blood pressure; DBP, diastolic blood pressure; PR, pulse rate; RR, respiratory rate; BT, body temperature; GCS, Glasgow coma scale.
